# The *Pseudomonas aeruginosa* DksA1 protein is involved in H_2_O_2_ tolerance and within-macrophages survival and can be replaced by DksA2

**DOI:** 10.1038/s41598-022-14635-7

**Published:** 2022-06-21

**Authors:** Alessandra Fortuna, Diletta Collalto, Veronica Schiaffi, Valentina Pastore, Paolo Visca, Fiorentina Ascenzioni, Giordano Rampioni, Livia Leoni

**Affiliations:** 1 Department of Science, University Roma Tre, Rome, Italy; 2 Department of Molecular and Cellular Biology “Charles Darwin”, University Roma Sapienza, Rome, Italy; 3 IRCCS Fondazione Santa Lucia, Rome, Italy

**Keywords:** Bacterial genetics, Bacteriology, Pathogens

## Abstract

In Gram-negative pathogens, the stringent response regulator DksA controls the expression of hundreds of genes, including virulence-related genes. Interestingly, *Pseudomonas aeruginosa* has two functional DksA paralogs: DksA1 is constitutively expressed and has a zinc-finger motif, while DksA2 is expressed only under zinc starvation conditions and does not contain zinc. DksA1 stimulates the production of virulence factors in vitro and is required for full pathogenicity in vivo*.* DksA2 can replace these DksA1 functions. Here, the role of *dksA* paralogs in *P. aeruginosa* tolerance to H_2_O_2_-induced oxidative stress has been investigated. The *P. aeruginosa dksA1 dksA2* mutant showed impaired H_2_O_2_ tolerance in planktonic and biofilm-growing cultures and increased susceptibility to macrophages-mediated killing compared to the wild type. Complementation with either *dksA1* or *dksA2* genes restored the wild type phenotypes. The DksA-dependent tolerance to oxidative stress involves, at least in part, the positive transcriptional control of both *katA* and *katE* catalase-encoding genes. These data support the hypothesis that DksA1 and DksA2 are eco-paralogs with indistinguishable function but optimal activity under different environmental conditions, and highlight their mutual contribution to *P. aeruginosa* virulence.

## Introduction

Bacteria constantly contend with toxic reactive oxygen species (ROS) such as hydrogen peroxide (H_2_O_2_), superoxide (O_2_^−^), hypochlorous acid (HOCl) and the hydroxyl free radical (^·^OH), which cause DNA damage, lipid peroxidation and negatively affect protein structure and functionality. These molecules are ubiquitous in the environment, and some of them are also generated by aerobically growing bacterial cells. During infection, pathogenic bacteria must cope with immune response cells, which produce particularly high levels of ROS that are either released in the extracellular milieu or used to kill engulfed bacteria^1^. After phagocytosis of bacteria, professional phagocytic cells, including macrophages, activate the NOX2 NADPH oxidase, a multicomplex enzyme assembled in the phagosomal membrane, which catalyses the reduction of molecular oxygen to superoxide. The latter can directly kill the bacteria within phagosomes and is converted to H_2_O_2_ by superoxide dismutases (SODs)^2,3^. Interestingly, bacterial SODs contribute to the production of microbicidal H_2_O_2_ within macrophages, as recently observed in *Pseudomonas aeruginosa*^4^. H_2_O_2_ can be further converted to hydroxyl free radical via Fenton reaction or halogenated ROS. This quick ROS production in the phagosome, a process known as oxidative burst, plays a key role in the host innate immune response^5,6^. Bacteria must also cope with extracellular ROS. As an example, the oxidases DUOX1 and DUOX2 generate H_2_O_2_ on the extracellular side of the apical membrane of the airway epithelium^7^.

*P. aeruginosa* is an opportunistic human pathogen responsible for life-threatening acute and chronic infections in immunocompromised, hospitalized, and cystic fibrosis patients^5^. The increasing prevalence of multi-drug resistant *P. aeruginosa* strains in the hospital setting increases the risk of antimicrobial treatment failure. Therefore, *P. aeruginosa* belongs to the ESKAPE group of bacterial pathogens (*Enterococcus faecium*, *Staphylococcus aureus*, *Klebsiella pneumoniae*, *Acinetobacter baumannii*, *P. aeruginosa*, *Enterobacter* spp.) for which novel treatment approaches are urgently needed^8,9^.

In addition to a wide arsenal of virulence factors, *P*. *aeruginosa* expresses an array of ROS-scavenging enzymes to counteract endogenous and exogenous ROS, including the four catalases KatA, KatB, KatE and KatN^5,6,10–12^. The KatA and KatB catalases are crucial for *P. aeruginosa* adaptation to the high levels of H_2_O_2_ encountered in the host^11,13,14^. Expression of *katA* is constitutive and increases in the presence of H_2_O_2_. Conversely, the expression of *katB* is induced only upon exposure to exogenous H_2_O_2_^10,15^. Up to now, it is unclear if also the KatE (also named KatC) and KatN (also named KatM) catalases are involved in H_2_O_2_ detoxification in *P. aeruginosa*.

The bacterial stringent response regulator DksA contributes to ROS tolerance in bacterial pathogens such as *Salmonella enterica* and *Haemophilus ducreyi*^16–19^. DksA also plays a key role in the control of virulence-related genes. In some pathogenic bacteria (*e.g., Escherichia coli*, *H. ducreyi*, *P. aeruginosa*, *S. enterica*, *Vibrio cholerae* and *A. baumannii*) *dksA* mutants are less virulent than the wild type both in vitro and in vivo^18,20–25^.

Up to now, *P. aeruginosa* is the only bacterium known to express two functional DksA paralogs, encoded by the *dksA1* and *dksA2* genes. The DksA1 and DksA2 proteins share a similar overall structure containing: a RNAP-interacting coiled-coil domain, a globular domain and a C-terminal α-helix. However, the globular domain of DksA1 contains a canonical zinc-finger structure, with a four-cysteine motif (4-Cys) for zinc binding, while the DksA2 globular domain has only two cysteine residues (2-Cys), and does not coordinate zinc^26,27^. The constitutively expressed DksA1 protein broadly affects the *P. aeruginosa* transcriptome, and positively controls virulence genes expression^23,24^. Conversely, DksA2 is exclusively expressed under conditions of zinc starvation^28^.

By artificially inducing DksA2 expression in Luria–Bertani Broth (LB), our group has recently shown that the DksA2 protein can replace DksA1 in the control of about sixteen hundred genes, including those involved in virulence and biofilm formation^24^. Only a small number of functionally unrelated genes seems to be exclusively controlled by each one of the two DksA paralogues under our artificial conditions^24^. Since the zinc-containing 4-Cys motif is required for the proper folding and functionality of *P. aeruginosa* DksA1, our study supports the previous hypothesis that the structural stability and functionality of DksA1 could be impaired in zinc-poor environments, where the zinc-free DksA2 protein could replace or adjuvate DksA1 function^26,28^.

A difference in the functionality of *P. aeruginosa* DksA1 and DksA2 proteins has also been proposed by Crawford et al.^19^, who showed that a *S. enterica* ∆*dksA* mutant complemented with the *P. aeruginosa dksA2* gene was less resistant to H_2_O_2_ and more susceptible to macrophage-mediated killing than the same strain complemented with the *P. aeruginosa dksA1* gene. Although intriguing, the differential role of DksA1 and DksA2 in oxidative stress tolerance has not yet been tested in *P. aeruginosa*.

The objective of this study has been to investigate the role of DksA1 in *P. aeruginosa* tolerance to H_2_O_2_ and macrophages-mediated killing, and to verify to which extent DksA2 can replace DksA1 function. Evidence is here provided that DksA1 is required for H_2_O_2_ tolerance in both planktonic and biofilm growing cells, and that this protein protects against macrophages-mediated killing. One of the mechanisms involved in DksA-dependent resistance to oxidative stress implies the positive control of *katA* and *katE* genes expression. Finally, in the homologous *P. aeruginosa* system, DksA2 fully replaced the protective function of DksA1 against oxidative stress, in contrast with previous observations in the heterologous *Salmonella* system^19^.

## Results

### DksA1 contributes to hydrogen peroxide tolerance, and DksA2 can replace DksA1 function

In *P. aeruginosa,* the *dksA1* gene is constitutively expressed while the *dksA2* gene is strongly repressed by Zur in the presence of available zinc^24,28^. To achieve comparable expression levels of the two *dksA* paralogs in a rich laboratory medium, such as LB, we previously generated a set of four *P. aeruginosa* PAO1 recombinant strains: the wild type strain (PAO1) and the *dksA1 dksA2* double mutant (∆*dksA1-2*), both carrying the pME6032 empty vector, and the ∆*dksA1-2* mutant carrying either pDksA1 or pDksA2. The latter are pME6032-derivative plasmids for isopropyl β-D-1-thiogalactopyranoside (IPTG)-inducible expression of *dksA1* and *dksA2*, respectively (Supplementary Table S1). As shown previously, the deletion of *dksA* paralogs or the presence of pME6032 derivatives or IPTG addition did not affect growth in LB. In addition, IPTG induction drove similar levels of *dksA* paralogs expression in PAO1 ∆*dksA1-2* carrying pDksA1 or pDksA2^24^. Here, the same experimental framework has been used to investigate the role of DksA1 and DksA2 in *P. aeruginosa* response to oxidative stress caused by H_2_O_2_.

**Figure 1:**
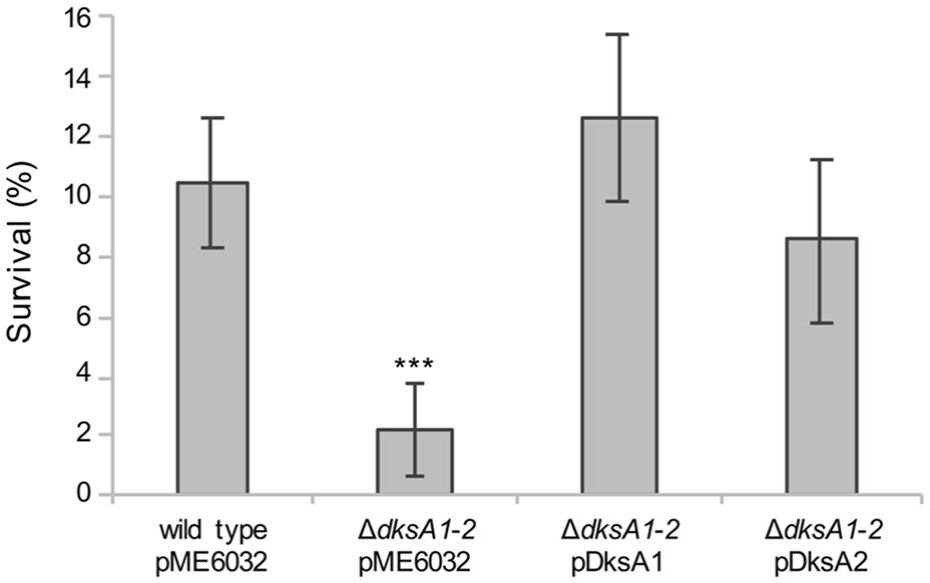
Effect of *dksA* paralogs on H_2_O_2_ tolerance in *P. aeruginosa* planktonic cultures. Survival of the indicated strains grown in LB supplemented with 0.1 mM IPTG to the late exponential phase (OD_600_ ≈ 2.5) after treatment with 50 mM H_2_O_2_ for 30 min. Survival is expressed as percentage of the CFU counts of the treated samples relative to the CFU counts of the corresponding untreated controls. The average values from five independent experiments are reported with standard deviations. ***p < 0.001 *versus* wild type (ANOVA).

The minimum inhibitory concentration (MIC) of H_2_O_2_ determined for planktonic *P. aeruginosa* cells in LB supplemented with 0.1 mM IPTG was 1 mM for all the tested strains. Subsequently, the *P. aeruginosa* tolerance to H_2_O_2_, *i.e*., the ability to survive transient exposure to 50X MIC H_2_O_2_ concentrations, was determined. Results indicate that the percentage of bacteria survival after 30-min treatment with 50 mM H_2_O_2_ was fivefold higher in PAO1(pME6032) than in the ∆*dksA1-2*(pME6032) mutant (Fig. 1). In addition, complementation with either *dksA1* or *dksA2* genes in Δ*dksA1-2*(pDksA1) and Δ*dksA1-2*(pDksA2) restored H_2_O_2_ tolerance, with no significant difference between the two *dksA* paralogs.

The role of *dksA1* and *dksA2* in *P. aeruginosa* biofilm tolerance to H_2_O_2_ was also investigated. To this purpose, the MIC of H_2_O_2_ was determined in M9-glu-CAA, a chemically-defined medium suited for *P. aeruginosa* biofilms studies^24^. In M9-glu-CAA, the MIC of H_2_O_2_ was 0.1 mM for all the tested strains, thus tenfold lower than that determined in LB. Hence, pre-formed *P. aeruginosa* biofilms were challenged with supra-MIC concentrations of H_2_O_2_, ranging from 50 to 200X MIC, and compared to the respective untreated controls.

**Figure 2:**
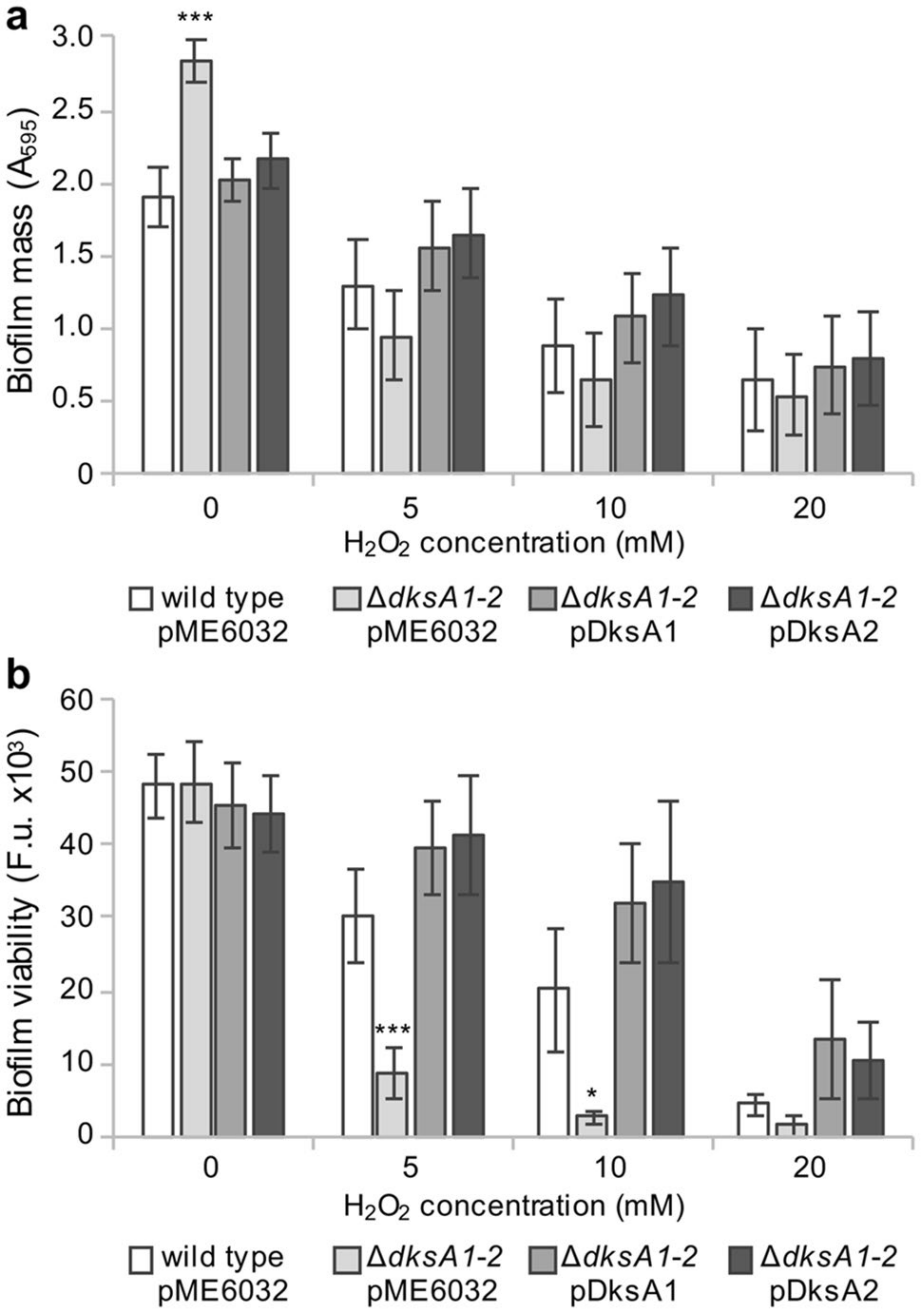
Effect of *dksA* paralogs on H_2_O_2_ tolerance in *P. aeruginosa* biofilm. Biofilm biomass (**a**) and viability (**b**; expressed as Fluorescence units, F. u.) of the indicated strains grown in M9-glu-CAA supplemented with 0.1 mM IPTG, challenged for 6 h with 5, 10 or 20 mM H_2_O_2_. The average values from five independent experiments performed on multiple wells per condition are reported with standard deviations. ***p < 0.001 versus untreated wild type (a; ANOVA); *p < 0.05 and ***p < 0.001 versus wild type challenged with 10 and 5 mM H_2_O_2_, respectively (**b**; ANOVA).

In the absence of H_2_O_2_ treatment, higher levels of biofilm biomass were detected by crystal violet (CV) staining in the ∆*dksA1-2*(pME6032) mutant compared to PAO1(pME6032) and the Δ*dksA1-2*(pDksA1) and Δ*dksA1-2*(pDksA2) complemented strains (Fig. 2a), in accordance with our previous study^24^. However, the fluorescein diacetate (FDA) assay showed that the metabolic activity of PAO1(pME6032) and ∆*dksA1*-*2*(pME6032) biofilms were comparable in the absence of H_2_O_2_ (Fig. 2b), suggesting that in the ∆*dksA1*-*2*(pME6032) mutant there could be an increased production of extracellular biofilm matrix components compared to PAO1(pME6032). After challenging with H_2_O_2_, a dose–response biofilm disrupting effect was detected for all the tested strains by using both CV and FDA detection methods, though ∆*dksA1*-*2*(pME6032) biofilms were significantly more reduced by the treatment compared to the wild type and complemented strains (Fig. 2). As observed in liquid cultures (Fig. 1), complementation with either *dksA1* or *dksA2* genes in the Δ*dksA1-2* mutant restored similar levels of H_2_O_2_ tolerance.

Overall, since *dksA2* expression is shut-off in PAO1 grown in the zinc-proficient media, such as LB and M9-glu-CAA^24^, the decreased H_2_O_2_ tolerance of the ∆*dksA1*-*2* mutant, relative to the PAO1 parent strain, should in principle be attributable to the lack of *dksA1* expression. Hence, the above results suggest that DksA1 is required for H_2_O_2_ tolerance of both planktonic and biofilm cultures of *P. aeruginosa*, and that *dksA2* can replace *dksA1* function when artificially expressed in zinc-containing media.

### DksA1 contributes to macrophages-mediated killing tolerance, and DksA2 can replace DksA1 function

As mentioned in the introduction, the oxidative burst is the first microbicidal mechanism activated by macrophages to kill engulfed bacteria^5,6^. Thus, we analysed the role of DksA1 and DksA2 in counteracting oxidative stress within macrophages. First, RAW macrophages were infected for 15 and 30 min with PAO1 or the ∆*dksA1-2* mutant, both carrying the pUCP30T-GFP*mut3* plasmid for constitutive expression of the green fluorescent protein (GFP). Flow cytofluorimetry analysis showed that the percentage of fluorescent macrophages was similar in both infections, revealing that the ∆*dksA1-2* double mutation has no impact on *P. aeruginosa* internalization by macrophages (Supplementary Fig. S1).

**Figure 3:**
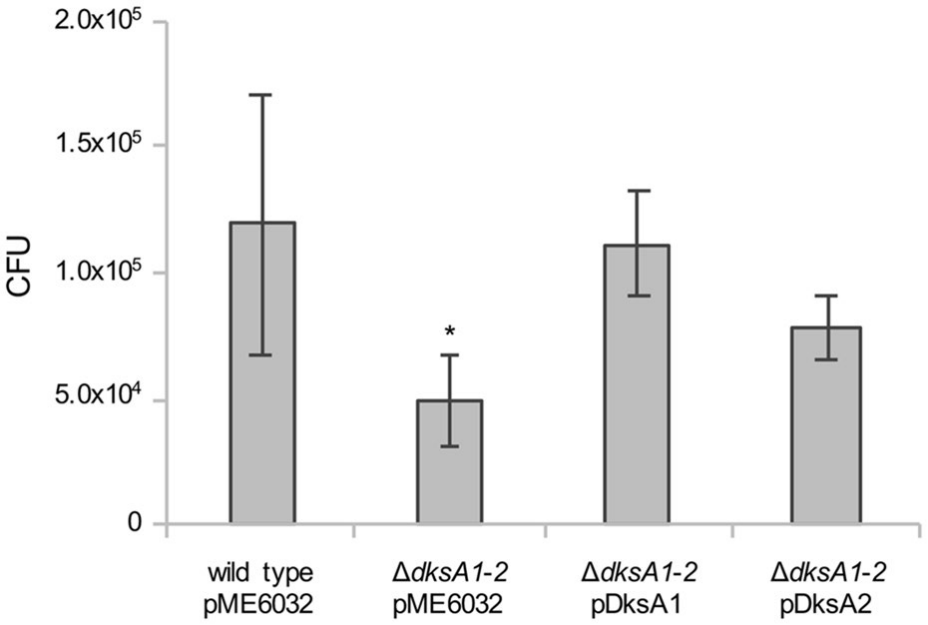
Intracellular survival of *P. aeruginosa* strains in RAW macrophages. Macrophages were infected for 30 min with the indicated strains, treated with gentamycin and lysed. Total live bacteria in cell lysates were recovered and quantified by CFU counts on PIA plates. The average CFUs per well from four independent experiments, each in duplicate, are reported with standard deviations. *p < 0.05 versus wild type (ANOVA).

Hence, the susceptibility to macrophages-mediated killing was compared in *P. aeruginosa* PAO1 and its isogenic recombinant strains. After 30 min from the infection, the number of live bacteria recovered from RAW macrophages was higher for PAO1(pME6032) than for the ∆*dksA1*-*2*(pME6032) mutant (Fig. 3). Taking into consideration that these strains are similarly phagocytosed, this result indicates that the ∆*dksA1*-*2* mutant is killed more efficiently than the wild type strain. The intra-macrophage survival defect of the ∆*dksA1*-*2* double mutant was complemented by pDksA1- or pDksA2-driven expression of either *dksA1* or *dksA2*, respectively (Fig. 3), providing evidence that DksA2 can replace DksA1 function even inside macrophages. Overall, these results indicate that both DksA paralogs can protect *P. aeruginosa* against NADPH-oxidase-dependent macrophages oxidative burst.

### DksA1 modulates endogenous ROS content by controlling catalases expression, and DksA2 can replace DksA1 function

**Figure 4:**
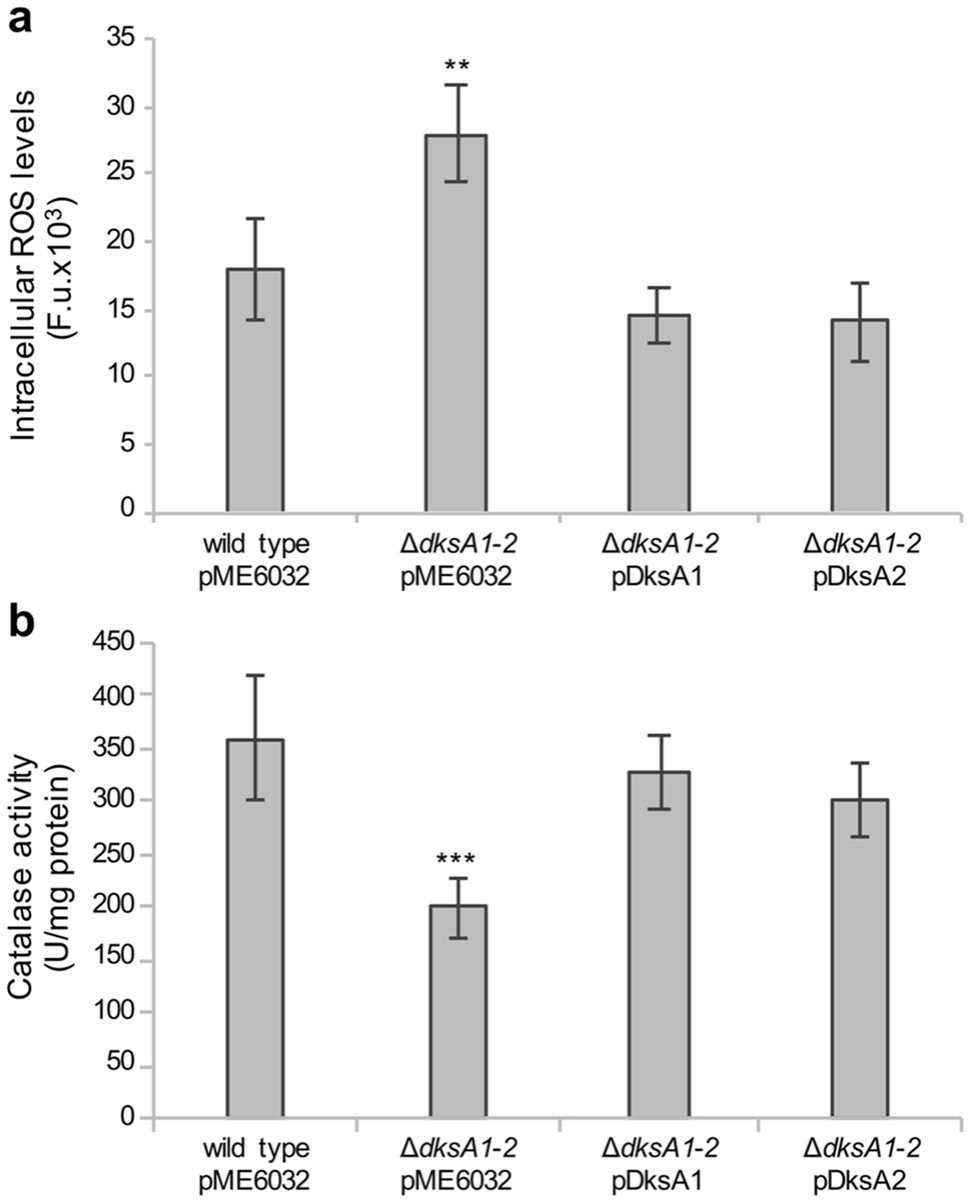
Effect of *dksA1* and *dksA2* on endogenous ROS levels and catalase activity. Intracellular levels of ROS (expressed as Fluorescence Units, F. u.) (**a**) and catalase activity (**b**) were determined in the indicated strains grown to the late exponential phase (OD_600_ ≈ 2.5) in LB supplemented with 0.1 mM IPTG. The average values from three independent experiments are reported with standard deviations. **p < 0.01, ***p < 0.001 versus wild type (ANOVA).

The results presented above show that both *P. aeruginosa dksA* paralogs restore wild type levels of tolerance to H_2_O_2_ exposure or macrophage oxidative burst. However, the DksA paralogs are also involved in the fine modulation of several central metabolic pathways, and an unbalanced metabolism could also affect endogenous ROS levels^29^. In agreement with this hypothesis, endogenous ROS levels were about 36% lower in PAO1(pME6032) than in ∆*dksA1*-*2*(pME6032), both grown in LB (Fig. 4a). Hence, DksA1 contributes to the homeostasis of the intracellular ROS levels naturally produced by cellular metabolism during aerobic growth, even in the absence of exogenous oxidative stress and nutrient starvation. An enzymatic assay revealed that ∆*dksA1*-*2*(pME6032) disclosed a 44% reduction of catalase activity compared to PAO1(pME6032) (Fig. 4b), suggesting that the increased ROS levels measured in the ∆*dksA1*-*2* mutant could be related, at least in part, to a decreased expression of the H_2_O_2_ detoxifying enzymes. Finally, the expression of either DksA1 or DksA2 in the Δ*dksA1-2*(pDksA1) and Δ*dksA1-2*(pDksA2) fully restored wild type catalase activity and ROS levels, providing evidence that also in this case DksA2 can replace DksA1.

To study the impact of the *dksA* gene products on the expression of different catalase-encoding genes, relative mRNA levels of *katA*, *katB*, *katE* and *katN* were investigated by means of Real Time PCR analyses in *P. aeruginosa* cultures grown in LB containing 0.1 mM IPTG, treated or not with 1 mM H_2_O_2_ (1X MIC) for 20 min, to measure the expression of the H_2_O_2_-inducible genes. Preliminary experiments showed that the expression of *dksA1* and *dksA2* was not affected in PAO1 in response to 1 mM H_2_O_2_ (Supplementary Fig. S2).

**Figure 5:**
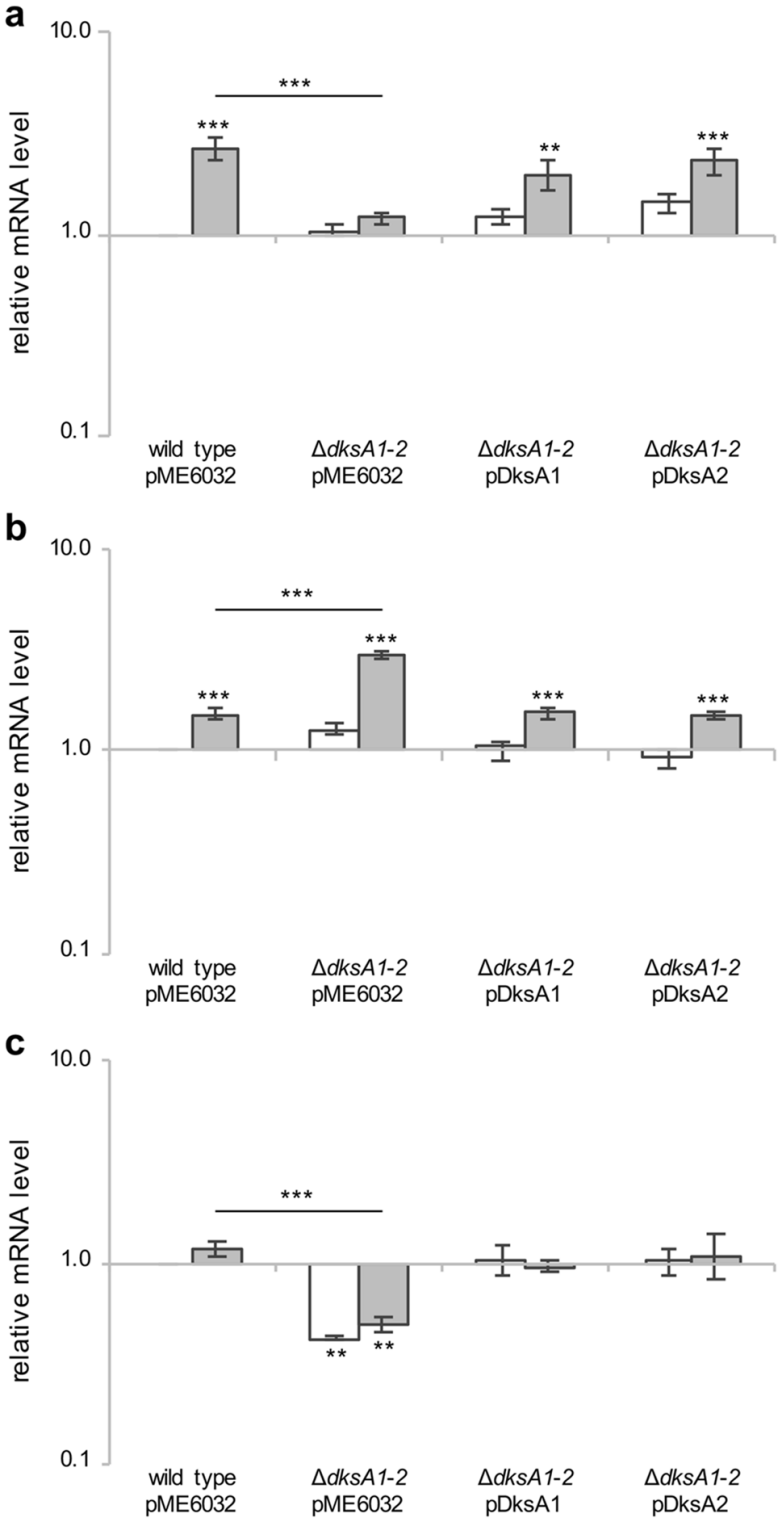
Expression analysis of oxidative stress response genes. Bacterial cultures were grown to the exponential phase (OD_600_ ≈ 2.5) in LB supplemented with 0.1 mM IPTG, then incubated with or without 1 mM H_2_O_2_ for 20 min. The mRNA levels of *katA* (**a**), *katB* (**b**) and *katE* (**c**) were determined by Real Time PCR analysis. Gene expression in the indicated untreated (white bars) or treated (grey bars) strains was reported as fold change in gene expression relative to the level of each gene in the untreated wild type PAO1(pME6032) strain. The average of two independent analyses, each performed on three technical replicates, is reported with standard deviations. Asterisks above the horizontal lines refer to *p* values versus wild type challenged with 1 mM H_2_O_2_; asterisks above the bars refer to *p* values *versus* the untreated wild type; **p < 0.01, ***p < 0.001 (ANOVA).

As previously reported^10,15^, the mRNA levels of both *katA* and *katB* increased in PAO1 exposed to H_2_O_2_ (Fig. 5). Interestingly, the *dksA1 dksA2* double deletion had opposite effect on the H_2_O_2_-dependent expression of these genes. Indeed, *katA* and *katB* mRNA levels decreased and increased in ∆*dksA1*-*2*(pME6032) relative to PAO1(pME6032) in the presence of H_2_O_2_, respectively (Fig. 5a,b).

Concerning the other two *P. aeruginosa* catalase-encoding genes, *katN* expression was not induced by H_2_O_2_ and not affected by the *dksA1 dksA2* double deletion (Supplementary Fig. S3). Conversely, *katE* mRNA levels were downregulated in ∆*dksA1*-*2*(pME6032) relative to PAO1(pME6032), irrespective of H_2_O_2_ presence (Fig. 5c).

Since DksA2 is expressed at low-basal levels in PAO1(pME6032) grown in LB, the above results show that at least DksA1 is required for the H_2_O_2_-dependent transcriptional upregulation and downregulation of *katA* and *katB*, respectively. In addition, DksA1 is also required for full expression of *katE*, irrespective of H_2_O_2_.

Finally, the IPTG-driven expression of either DksA1 or DksA2 in the Δ*dksA1-2*(pDksA1) and Δ*dksA1-2*(pDksA2) strains restored wild type mRNA levels for all the tested genes (Fig. 5), demonstrating that DksA2 can replace the function of DksA1 in promoting *katA* and *katE* expression, and in down-regulating *katB* expression.

## Discussion

The stringent response controls bacterial adaption to nutrient starvation and other stressful conditions. In Gram-negative bacteria, (p)ppGpp binds the ß’ subunit of RNAP at the interface with the ω subunit and in a second site, sandwiched between the ß′ subunit and the DksA protein, which is strictly required for this interaction^30,31^. Hence, both (p)ppGpp and DksA are required for full stringent response, even if the two factors can also work independently^20,32–35^. While previous studies showed that (p)ppGpp positively affects *P. aeruginosa* tolerance to H_2_O_2_^36,37^, the contribution of DksA1 and DksA2 to this process remained unknown so far.

In the culture media used in this and our previous study^24^, *dksA2* expression is strongly repressed by zinc in the *P. aeruginosa* PAO1 wild type strain. Hence, the comparison of the wild type and *dksA1 dksA2* double mutant phenotypes only reveals the effect of DksA1 depletion. However, complementation of the double mutant with either DksA1 or DksA2 expressed by the corresponding gene under the control of an IPTG-inducible promoter allows comparing the activity of the two DksA paralogs. Overall, the *dksA1* and *dksA2* expression levels obtained by this approach are adequate to restore wild type levels of all the tested phenotypes.

While the *dksA1 dksA2* double deletion does not affect *P. aeruginosa* long-term exposure to H_2_O_2_ in both LB and M9-glu-CAA (i.e., the MIC of H_2_O_2_ is identical for wild type PAO1 and the Δ*dksA1-2* mutant), DksA1 increases *P. aeruginosa* tolerance to transient H_2_O_2_ exposure, both in planktonic and in biofilm growing cultures. In line with these results, the deletion of both *dksA1* and *dksA2* undermines *P. aeruginosa* survival within macrophages. The *dksA1 dksA2* double deletion leads to an increase in *P. aeruginosa* biofilm biomass, as already shown in our previous study by using different biofilm models (i.e., CV staining, pellicle formation and Congo-Red binding assays)^24^. In the same study, it was shown that c-di-GMP levels were lower in the Δ*dksA1-2* mutant compared to the wild type, indicating that DksA1 negatively controls biofilm formation through a c-di-GMP-independent pathway. Furthermore, the RNA-Seq analysis showed that some of the known *P. aeruginosa* biofilm-related genes were up-regulated and others were down-regulated in the Δ*dksA1-2* mutant compared to the wild type strain^24^. This study showed that the biofilm cells of the Δ*dksA1-2* mutant are less viable after H_2_O_2_ exposure relative to wild type biofilm cells, despite the increased biofilm biomass. This suggests a defect in the Δ*dksA1-2* biofilm matrix composition and development. In the future, it could be interesting to further investigate this issue.

In the absence of exogenous H_2_O_2_, the Δ*dksA1-2* mutant showed an increase in the endogenous ROS content and a decrease in the overall catalase activity, compared to wild type PAO1. This could be due to the reduced expression of *katE* in the Δ*dksA1-2* strain, since the expression of the other *P. aeruginosa* catalase genes was not affected by *dksA1 dksA2* double deletion in the absence of oxidative stress. However, since DksA1 controls more than one thousand genes, including primary metabolism pathways^23,24^, the ROS increase measured in the Δ*dksA1-2* strain could also be due to a generalized metabolic effect. Moreover, in the presence of exogenous H_2_O_2_, *katA* and *katE* genes were downregulated while *katB* was up-regulated in the double mutant compared to the wild type PAO1.

Overall, it is plausible that the defective expression of *katA* and *katE* genes, combined with an altered metabolism leading to increased levels of endogenous ROS, could overcome the effects of the increased *katB* expression, ultimately reducing the tolerance of the Δ*dksA1-2* mutant to exogenous H_2_O_2_ and macrophages-mediated killing, relative to wild type PAO1. In this context, it is to note that the role of the KatE catalase in *P. aeruginosa* H_2_O_2_ tolerance has not been clarified yet. Indeed, KatE seems not to be involved in H_2_O_2_ detoxification in the PA14 strain^11^, while a protective role of KatE against oxidative stress only at elevated temperatures was described in the PAO6049 strain^38^.

Interestingly, a *P. aeruginosa* mutant lacking the *relA* and *spoT* genes, required for (p)ppGpp synthesis, showed several phenotypes similar to those caused by the *dksA1 dksA2* double deletion, i.e., increased susceptibility to H_2_O_2_, impaired overall catalase activity and high levels of endogenous ROS with respect to the parental strain^36,37,39^. However, different from what we observed for DksA1, (p)ppGpp positively controls the expression of both *katA* and *katB* genes*.* Interestingly, this (p)ppGpp-dependent regulation is independent of exogenous H_2_O_2_ for *katA* and H_2_O_2_-dependent for *katB*^36,37^.

By combining our data with previous findings, it can be argued that both (p)ppGpp and DksA1 play a major role in *P. aeruginosa* tolerance to endogenous and exogenous oxidative stress, even if they differently regulate the expression of H_2_O_2_-scavenger enzymes. Further studies should be carried out to unravel the regulatory mechanisms underlying the different effects of DksA1 and (p)ppGpp on *katA*, *katB* and *katE* genes expression, as well as the impact of KatE-mediated H_2_O_2_ detoxification in *P. aeruginosa* PAO1.

To our knowledge, the importance of DksA in oxidative stress tolerance has so far been documented only for *S. enterica* and *H*. *ducreyi*. Each one of these pathogens expresses only one DksA protein, containing a zinc finger domain, as it is for DksA1^16,18^. Hence the possibility that DksA2 could replace DksA1 deserves special attention, also considering that a previous study showed that a *S. enterica ∆dksA* mutant expressing the *P. aeruginosa dksA2* gene had an impaired tolerance to H_2_O_2_ in vitro, reduced survival in macrophages, and reduced virulence in mice relative to the *S. enterica ∆dksA* mutant expressing the *P. aeruginosa dksA1* gene. The different behaviour of DksA1 and DksA2 in the heterologous host *Salmonella* was supported by experiments carried out with the purified proteins, showing that DksA1 was more resistant to oxidation than DksA2, likely due to the presence of zinc in the globular domain^19^*.* Different from what observed in *S. enterica*, we showed by multiple experimental strategies that DksA2 replaces all DksA1 functions related to *P. aeruginosa* H_2_O_2_ tolerance when expressed in its isogenic background, at least under our experimental conditions.

Certainly, the possibility that *dksA2* overexpression may mask a higher sensitivity of DksA2 to ROS than DksA1 cannot be rule out, and this issue should be further studied under conditions of zinc starvation, mimicking the environmental conditions where *dksA2* is naturally expressed. In the wild type genetic background, under zinc-limiting conditions, both *dksA1* and *dksA2* genes are expressed from their natural promoters^28^ and they may have a different impact on *P. aeruginosa* oxidative stress response. However, while *dksA2* expression levels are related to the extent of zinc limitation, DksA1 activity might be impaired under conditions of extreme zinc deficiency, due to incorrect folding of the Zn-finger domain^28^.

Zinc depletion is recognised as an innate immunity mechanism faced by pathogens during the infection. Interestingly, *dksA2* is expressed in the sputum of cystic fibrosis patients colonized by *P. aeruginosa*^40,41^, supporting a role for DksA2 at least in this kind of infection. While further studies should be carried out to better define the relative importance of DksA1 and DksA2 in the protection from H_2_O_2_ exposure under zinc starvation conditions, this study provides the evidence that DksA2 has the intrinsic ability to replace the *dksA1* gene product under oxidative stress conditions.

Overall, the results of this study are in line with our previous observation that DksA2 can replace DksA1 function in controlling the expression of almost all the DksA1-regulated genes and DksA1-dependent virulence phenotypes^24^ strengthening the hypothesis that DksA1 and DksA2 are eco-paralogs as defined by Sanchez-Perez et al.^42^, i.e., paralogs with the same overall function, having optimal activity under different environmental conditions.

## Materials and methods

### Bacterial strains and growth conditions

Bacterial strains used in this study are listed in Supplementary Table S1. *P. aeruginosa* strains were routinely grown at 37 °C in shaking conditions in LB^43^, or LB supplemented with 1.5% (w/v) agar. When required, the media were supplemented with 0.1 mM IPTG or 100 µg/mL tetracycline (Tc). M9 medium [0.77% (w/v) Na_2_HPO_4_·2H_2_O; 0.3% (w/v) KH_2_PO_4_; 0.05% (w/v) NaCl; 0.025% (w/v) MgSO_4_·7H_2_O; 0.002% (w/v) CaCl_2_]^43,44^ supplemented with 0.39% (w/v) glucose and 0.5% (w/v) casamino acids (CAA) as carbon sources (M9-glu-CAA)^24^ was used for specific assays. H_2_O_2_ [stock 30% (v/v), Sigma Aldrich] was added to the media at the concentrations indicated in the text.

### MIC assays

The MIC of H_2_O_2_ was evaluated as previously detailed^45^ with the standard microdilution method, according to the Clinical and Laboratory Standards Institute guidelines^46^. Cultures of *P. aeruginosa* were incubated at 37 °C with shaking in LB containing 100 μg/mL Tc or in M9-glu-CAA containing 50 μg/mL Tc, both supplemented with 0.1 mM IPTG. After overnight growth, cultures were diluted to an optical density at 600 nm wavelength (OD_600_) of ≈ 0.0005 (ca. 5 × 10^5^ CFU/mL) in 100 μL of LB or M9-glu-CAA, both supplemented with 0.1 mM IPTG, in the presence of increasing concentrations of H_2_O_2_. The MIC values were evaluated after 24 h of static incubation at 37 °C.

### H_2_O_2_ killing assays

H_2_O_2_ killing assays were performed as previously described^36^, with minor modifications. Late exponential phase cultures (OD_600_ ≈ 2.5) were normalized to an OD_600_ of 0.5 in LB supplemented with 0.1 mM IPTG and 50 mM H_2_O_2_ before incubation at 37 °C in shaking conditions. At the same time, untreated cultures were incubated at 37 °C as a control. After 30 min, 0.2% (w/v) sodium thiosulfate was added to neutralize residual H_2_O_2_. Colony forming units (CFU) were determined by the standard microdilution technique on LB agar plates. Survival was reported as percentage of CFU counted in the treated sample relative to the CFU counted in the untreated sample.

### Biofilm assays

The crystal violet (CV) binding assay and the fluorescein diacetate (FDA) assay were performed in microtiter plates as already detailed in previous studies^47–49^, with slight changes. Bacterial cells were grown in M9-glu-CAA with 50 µg/mL Tc and 0.1 mM IPTG for 8 h in shaking conditions and subsequently diluted to OD_600_ ≈ 0.015 in fresh M9-glu-CAA medium supplemented with 0.1 mM IPTG. Aliquots of 100 µL were transferred to sterile 96-well polystyrene microtiter plates and incubated at 30 °C for 15 h in static conditions. After the removal of the planktonic phase, biofilms were refreshed with 100 μL of fresh medium supplemented with 5, 10 or 20 mM H_2_O_2_ (treated biofilms) or without the addition of H_2_O_2_ (untreated biofilms). Microtiter plates were incubated at 30 °C for 6 h. After removing the liquid phase, biofilm mass was quantified by CV binding assay, while biofilm viability was determined by FDA assay. Briefly, CV binding assay was performed staining the attached cells with 1% (w/v) CV for 15 min. Hence, all wells were washed four times with distilled water and air dried. Finally, the biofilm-bound dye was solubilized with 200 µL of ethanol for 10 min and measured as A_595_ in an automated luminometer-spectrometer plate reader (Tecan Spark 10 M). To perform the FDA assay, the FDA stock solution (10 mg/mL, prepared in acetone; Sigma Aldrich) was diluted 1:100 in 100 mM 3-(*N*-morpholino)-propanesulfonic acid (MOPS, pH 7.0; FDA working solution) and 200 μL of FDA working solution were dispensed in all wells. The microtiter was incubated in the dark at 37 °C in static condition. Fluorescence was measured at 485 nm excitation and 535 nm emission wavelengths after 1 h through an automated luminometer-spectrometer plate reader (Tecan Spark 10 M).

### Macrophages infection assays

Murine macrophages RAW264.7 (ATCC TIB71) were grown in Dulbecco’s modified Eagle’s medium (DMEM; Corning) high glucose supplemented with 10% (v/v) fetal bovine serum and 1% (w/v) glutamine (all from EuroClone, Italy) at 37 °C in 5% CO_2_. For the infection assay, the day before infection 5 × 10^4^ cells/well were seeded in 48-well plates and incubated over-night in antibiotic-free medium. Each well was infected with different PAO1 strains at a multiplicity of infection (MOI) of 10. After 30 min of incubation at 37 °C, extracellular bacteria were killed by gentamycin (600 µg/mL) treatment during 15 min of incubation. For counting intracellular bacteria, infected RAW264.7 cells were washed with phosphate-buffered saline (PBS), lysed with 1% (v/v) Triton X-100, and dilutions plated on *Pseudomonas* isolation agar (PIA).

For the phagocytosis assay, macrophages were infected as described above, using GFP expressing bacteria obtained by transformation of PAO1 and ∆*dksA1*-*2* with the pUCP30T-GFP*mut3* plasmid (Supplementary Table S1)^50^. After either 15 or 30 min of infection, infected macrophages were washed twice with PBS, detached from the wells, recovered by centrifugation and finally resuspended in 300 µL PBS for flow cytometry (BD FACSCalibur, BD Biosciences, France). Phagocytosis was evaluated by the fraction of GFP positive cells in the bulk populations. Data were analysed using the CellQuest software and images processed using FlowJo.

### Measurement of intracellular ROS levels

The intracellular ROS levels were measured as previously described^51^, with minor modifications. *P. aeruginosa* strains were grown in LB with 0.1 mM IPTG to the late exponential phase (OD_600_ of ≈ 2.5). Then, cells were collected by centrifugation, washed with PBS, and resuspended in PBS at an OD_600_ of about 1.25. Bacteria were incubated with 10 µM 2′,7′-dichlorodihydrofluorescein diacetate (H_2_DCFDA; stock 2 mM, dissolved in dimethyl sulfoxide [DMSO]; Sigma Aldrich) for 20 min at 37 °C in the dark. Cells treated with an equal volume of DMSO were used as a negative control. Aliquots (200 µL) of each bacterial suspension were dispensed in a 96-well black microtiter plate. Fluorescence was measured at 485 nm excitation and 535 nm emission wavelengths with an automated luminometer-spectrometer plate reader (Tecan Spark 10 M) and normalized to the OD_600_ of each sample.

### Catalase activity assay

Catalase activity was measured as described previously^52^, with minor modifications. Bacteria were grown in LB with 0.1 mM IPTG to the late exponential phase (OD_600_ of ≈ 2.5). Hence, cells were collected by centrifugation, resuspended in 50 mM potassium-phosphate buffer (PPB, pH 6.8), lysed by sonication and subsequently centrifugated to collect the supernatants. Total proteins from the soluble fraction were quantified by the Bradford assay^53^, with bovine serum albumin as the standard. Aliquots containing 10 µg of proteins were added to 50 mM PPB, and catalase activity was monitored by following the decomposition of 20 mM H_2_O_2_ in 50 mM PPB at 240 nm (OD_240_) for 1 min (readings every 10 s) by using UV-transparent disposable cuvettes. One unit of catalase activity corresponded to 1 μmol of H_2_O_2_ hydrolysed *per* min by 1 mg of total proteins at 25 °C.

### RNA extraction and expression profiling experiments

*P*. *aeruginosa* strains were grown at 37 °C with shaking at 200 rpm in 10 mL of LB supplemented with 0.1 mM IPTG until reaching an OD_600_ of ≈ 2.5. Exponential phase cells were incubated with or without 1 mM H_2_O_2_ for 20 min in shaking conditions and 1 mL of each culture was mixed with 2 mL of RNA Protect Bacteria Reagent (Qiagen) for RNA extraction. Total RNA was extracted as previously described^24,54^. Briefly, RNA isolation was performed using RNeasy Mini Kit (Qiagen), including the on-column DNase I digestion step, followed by 1 h treatment at 37 °C with TURBO DNase (0.2 U per μg of RNA; Ambion) and SUPERase-In (0.4 U per µg of RNA; Ambion), and subsequent purification with the RNeasy Column Purification Kit (Qiagen). After confirmation of the lack of contaminating chromosomal DNA by PCR with the primer pair FWP*pqsL* and RVP*pqsL* (Supplementary Table S2), 1 μg of total RNA was used to synthesize cDNA using the iScript Reverse Transcription Supermix for RT-qPCR kit (BioRad). Real Time PCR analyses were performed using the iTaq Universal SYBR Green Supermix (BioRad), the AriaMx Real-Time PCR system (Agilent Technologies; software version 1.0) and the target-specific primers obtained by means of the Primer-Blast designing tool (www.ncbi.nlm.nih.gov/tools/primer-blast; Supplementary Table S2). The thermal cycling protocol was denaturation for 2 min at 95 °C, followed by 40 cycles of amplification at 95 °C for 15 s and 60 °C for 45 s. Fluorescence was registered in the last 15 s of the 60 °C step. The relative fold change in gene expression was calculated by the 2^−ΔΔCt^ method using *rpoD* as the housekeeping gene. The average data and standard deviations (SD) were calculated from two independent experiments each performed on three technical replicates.

### Statistical analysis

Statistical analysis was performed with the software GraphPad Prism 6.01, using one-way analysis of variance (ANOVA) followed by Tukey–Kramer multiple comparison test. Statistical analysis of macrophages infections used two-way ANOVA. A *p* value of < 0.05 was considered statistically significant.

## Supplementary Information

Supplementary Information.

## Data Availability

All data generated or analysed during this study are included in this published article (and relative Supplementary Information file).
