# Supplementary material for: The Pseudomonas aeruginosa DksA1 protein is involved in H2O2 tolerance and within-macrophages survival and can be replaced by DksA2
Source: Sci Rep. 2022 Jun 21;12:10404. doi: 10.1038/s41598-022-14635-7 (PMC9213440; doi:10.1038/s41598-022-14635-7)
Supplement: Supplementary file 1 — Supplementary Information. [file 41598_2022_14635_MOESM1_ESM.pdf]

The *Pseudomonas aeruginosa* DksA1 protein is involved in H_2O_2 tolerance and within-macrophages survival and can be replaced by DksA2

Alessandra Fortuna[1], Diletta Collalto[1], Veronica Schiaffi[2], Valentina Pastore[2], Paolo Visca[1,3], Fiorentina Ascenzioni[2], Giordano Rampioni[1,3], Livia Leoni[1*]

[1]Department of Science, University Roma Tre, Rome, Italy; [2]Department of Molecular and cellular biology "Charles Darwin", University Roma Sapienza, Rome, Italy; [3]IRCCS Fondazione Santa Lucia, Rome, Italy.

SUPPLEMENTAL MATERIAL

Figure S1. Effect of *dksA1 dksA2* double deletion on phagocytosis

Figure S2. Expression analysis of *dksA1* and *dksA2* in response to H_2O_2

Figure S3. Expression analysis of *katN*

Table S1. Bacterial strains used in this study

Table S2. Oligonucleotides used in this study

Figure S1

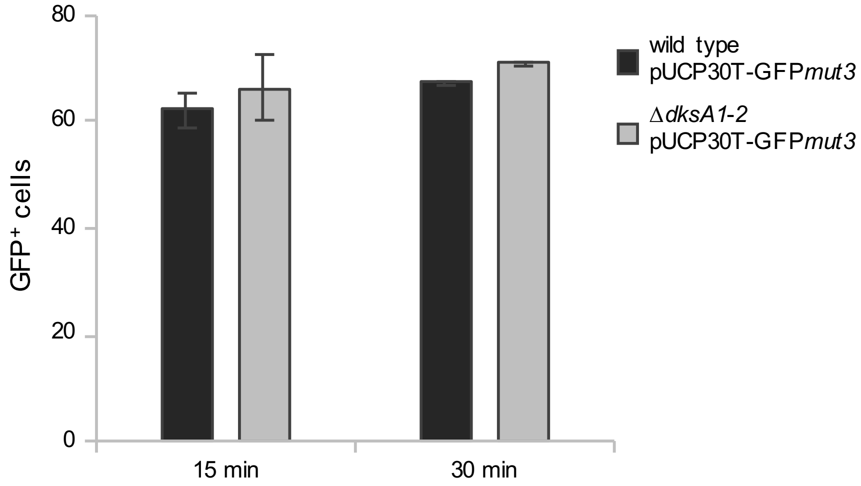

Figure S1. Effect of *dksA1 dksA2* double deletion on phagocytosis. Fluorescent macrophages in cell populations infected with the indicated GFP-expressing strains for 15 or 30 min. The average of two independent experiments, each in duplicate, is shown with standard deviations.

Figure S2

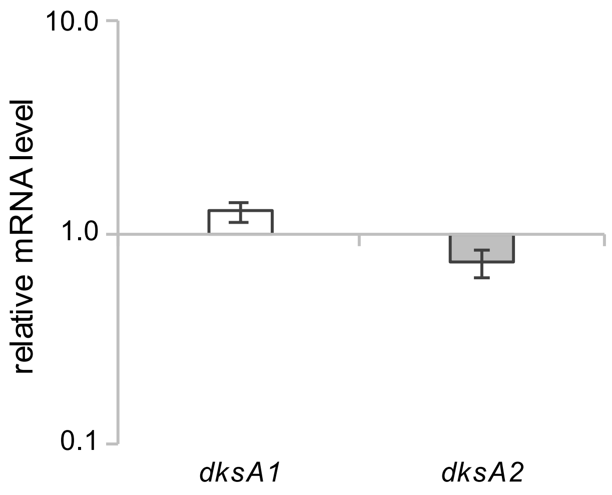

Figure S2. Expression analysis of *dksA1* and *dksA2* in response to H_2O_2. Real Time PCR analysis showing the relative mRNA level of the *dksA1* and *dksA2* genes. *P. aeruginosa* PAO1 grown to the exponential phase ($OD_{600} \approx 2.5$) in LB with 0.1 mM IPTG was challenged with 1 mM H_2O_2 for 20 min. Gene expression is reported as fold change relative to the untreated culture. The average of two independent analyses, each performed on three technical replicates, is shown with standard deviations.

Figure S3

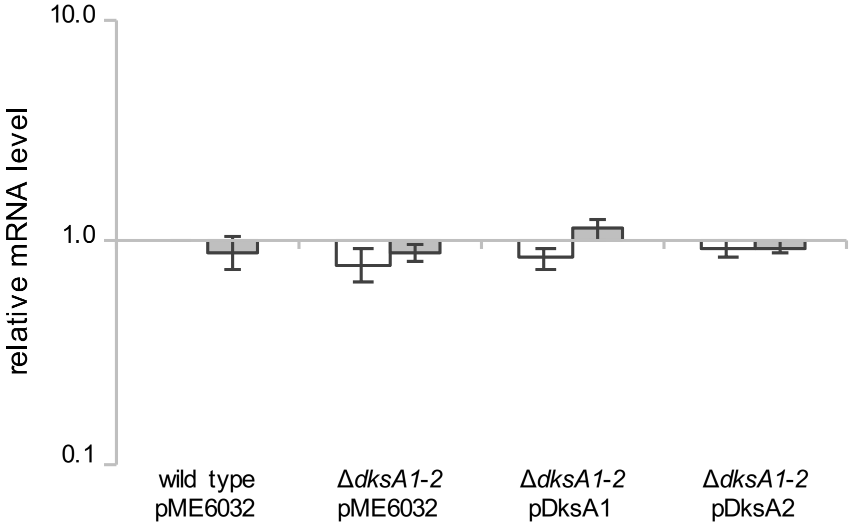

Figure S3. Expression analysis of *katN*. Bacterial cultures were grown to the exponential phase ($OD_{600} \approx 2.5$) in LB supplemented with 0.1 mM IPTG and then incubated with or without 1 mM H_2O_2 for 20 min. The mRNA levels of *katN* gene were determined by Real Time PCR analysis. Gene expression in the indicated untreated (white bars) or treated (grey bars) strains is reported as fold-change in gene expression relative to the untreated wild type PAO1(pME6032) strain. The average of two independent analyses, each performed on three technical replicates, is shown with standard deviations.

Table S1. Bacterial strains used in this study.

Strain	Relevant characteristics	Reference/Source
Pseudomonas aeruginosa		
PAO1(pME6032)	Wild type strain (ATCC 15692) containing the pME6032 expression vector.	[24]
PAO1(pUCP30T-GFP*mut3*)	Wild type strain (ATCC 15692) containing the pUCP30T-GFP*mut3* vector for the GFP constitutive expression [50].	This study
PAO1 Δ*dksA1-2*(pME6032)	PAO1 derivative strain carrying a deletion of the *dksA1* and *dksA2* genes and containing the pME6032 expression vector.	[24]
PAO1 Δ*dksA1-2*(pUCP30T-GFP*mut3*)	PAO1 derivative strain carrying a deletion of the *dksA1* and *dksA2* genes and containing the pUCP30T-GFP*mut3* vector for the GFP constitutive expression [50].	This study
PAO1 Δ*dksA1-2*(pDksA1)	PAO1 derivative strain carrying a deletion of the *dksA1* and *dksA2* genes and containing a pME6032 derivative for the IPTG-dependent expression of *dksA1*.	[24]
PAO1 Δ*dksA1-2*(pDksA2)	PAO1 derivative strain carrying a deletion of the *dksA1* and *dksA2* genes and containing a pME6032 derivative for the IPTG-dependent expression of *dksA2*.	[24]

Table S2. Oligonucleotides used in this study.

Name	Sequence (5'->3') [a]	Restriction site [b]
FWP*pqsL*	TCCGCTCGAGGATCGTCACCGTCAACTG	XhoI
RVP*pqsL*	TAACTGCAGCGTCATGGATGAGTCTCCG	PstI
FW*dksA1*RT	TTCACCGCGATCCTGAACAA	-
RV*dksA1*RT	ATCAGTTGCAGGGTCTCGTC	-
FW*dksA2*RT	AAGCCCAGCAGGACTTCTTC	-
RV*dksA2*RT	TGTCGAGCAGCTTCTTCTCC	-
FW*katA*RT	ATGCGTTTCTACACCGAGCA	-
RV*katA*RT	ATGGTCAACTGATGCAGCGA	-
FW*katB*RT	TGTATTCCAACGAGGGCACC	-
RV*katB*RT	GCTCACCAGGTCATTGGTCA	-
FW*katE*RT	GAGAACCAGCAGGACAGCTT	-
RV*katE*RT	GGGTCGTTGGTGAAGTCGAT	-
FW*katN*RT	ACTCGCATATCACCTCGCTG	-
RV*katN*RT	AAGGTCAGCGCATCCTTGAT	-
FW*rpoD*RT	CCACCGCCGAACTCACC	-
RV*rpoD*RT	CTCGTCGGTCTCGTGGTT	-

[a] The restriction sites included in the oligos are underlined.

[b] -, no restriction site introduced.
